# Non-linear association between lactate and 28 days mortality in elderly patients with sepsis across different SOFA score groups: results from the eICU Collaborative Research Database

**DOI:** 10.3389/fmed.2025.1605319

**Published:** 2025-07-03

**Authors:** Lanlang Zhang, Huiwen Liu, Dan Zhang, Youyou Deng, Xinglin Chen, Luyang Zhang

**Affiliations:** ^1^Department of Hemodialysis, Fuyong People’s Hospital of Baoan District, Shenzhen, China; ^2^Department of Epidemiology and Biostatistics, Empower U, X&Y Solutions Inc., Boston, MA, United States; ^3^Department of Hematology and Oncology, Shenzhen Children’s Hospital, Shenzhen, China

**Keywords:** lactate, mortality, elderly, sepsis, SOFA score

## Abstract

**Purpose:**

This study aimed to examine the correlation between lactate levels and 28 days mortality in elderly sepsis patients across different Sequential Organ Failure Assessment (SOFA) score groups following ICU admission.

**Methods:**

A multi-center retrospective cohort study utilized data from the eICU Collaborative Research Database, encompassing elderly sepsis patients from 208 ICUs across the United States during 2014–2015. Lactate levels and SOFA scores at admission were collected, with a focus on 28 days mortality post-ICU admission. A two-piece-wise linear regression model was developed to assess the threshold effects of lactate on outcomes and its variation across SOFA score categories. Smooth curve fitting was utilized.

**Results:**

Of the 5,150 patients with a median age of 76 years, 711 (13.8%) died within 28 days of ICU admission. A positive correlation was noted between lactate levels and mortality when lactate was < 3.7 mmol/l, with an adjusted odds ratio (OR) of 1.33 (95% CI: 1.17–1.51, *P* < 0.0001) for each increment in lactate. For lactate levels ≥ 3.7 mmol/L, mortality increased with an adjusted OR of 1.11 (95% CI: 1.05–1.18, *P* = 0.0003) for each increment in lactate. Moreover, mortality was low and rose gradually with increasing lactate levels in the SOFA score ≤ 5 group. Conversely, in the SOFA score > 5 group, mortality increased significantly, particularly when lactate levels exceeded 5 mmol/L.

**Conclusion:**

This study reveals a non-linear positive relationship between lactate and 28 days mortality among elderly sepsis patients. Furthermore, stratification by SOFA score demonstrated that patients with higher scores exhibited a heightened risk of mortality as lactate levels increased.

## Introduction

Sepsis is defined as a systemic inflammatory response to infection (1). In-hospital mortality for sepsis patients can exceed 10% (2). In 2017, there were approximately 48.9 million global sepsis cases, with a mortality rate of 19.7% (3). The aging population has contributed to an increase in sepsis cases among the elderly (4). Compared to younger adults, the elderly are more susceptible to comorbidities, chronic diseases, and reduced functional reserves, increasing their mortality risk (5). Furthermore, elderly patients may exhibit non-specific symptoms of sepsis response, which can impede the timely diagnosis of sepsis (6). It is imperative to acknowledge the significance of certain biomarkers in facilitating the diagnosis and risk stratification of sepsis in elderly patients (7).

Lactate, a metabolite produced through glycolysis (8), is recognized as a crucial biomarker for assessing disease severity and predicting mortality in critically ill patients (9). It is particularly important in conditions such as sepsis (10), acute cardiac conditions (11), liver cancer (12) and acute pancreatitis (13).

Lactate accumulation indicates tissue hypoperfusion in septic patients (2). Utilizing the Medical Information Mart for Intensive Care-IV database, a study showed that 28 days mortality for elderly septic patients reached 32.22%. Additionally, for every 1 mmol/L increase in lactate, the risk of 28 days mortality increased by 23% (10). The importance of the Sequential Organ Failure Assessment (SOFA) score in quantifying organ dysfunction necessary for diagnosing sepsis has been emphasized (2), and research suggests that incorporating lactate levels into the SOFA scoring system can improve the prediction of in-hospital mortality for elderly patients in intensive care units (14). Assessing lactate levels in different SOFA score groups among elderly septic patients is clinically significant, aiding in prognostic predictions and informing clinical decisions (10). Despite extensive research on the correlation between lactate and mortality in septic patients, studies focusing on lactate and 28 days mortality among elderly patients with sepsis across different SOFA score groups are scarce. The 28 days mortality is widely recognized as an important indicator for assessing short-term prognosis in septic patients, reflecting the immediate impact of acute illness (15, 16). Our study aimed to identify the lactate threshold at which 28 days mortality risk significantly increases and to examine the influence of the SOFA score on this correlation. We hypothesized that elevated lactate levels in elderly sepsis patients with higher SOFA scores would correlate with an elevated risk of 28 days mortality post-ICU admission. To assess this, a multi-center retrospective cohort study was conducted using data from the eICU Collaborative Research Database (eICU-CRD), covering 208 ICUs across the United States from 2014 to 2015.

## Materials and methods

### Data source

The data for this study were obtained from the eICU Collaborative Research Database (eICU-CRD), which encompasses over 200,000 ICU admissions from 208 hospitals across the United States during the period from 2014 to 2015. Data were automatically recorded through the Philips Healthcare eICU program and retrieved electronically for research purposes. The eICU-CRD is widely utilized in observational studies and has been validated for various clinical investigations (17, 18). Data access was granted by the institutional review boards of the Massachusetts Institute of Technology (MIT), Cambridge, MA, United States. The access was secured by Xinglin Chen, who performed data extraction (certification number: 40859994). All study procedures complied with the ethical guidelines and regulations of the eICU-CRD. As the study was retrospective and involved de-identified data, informed consent was waived.

### Study population

**FIGURE 1:**
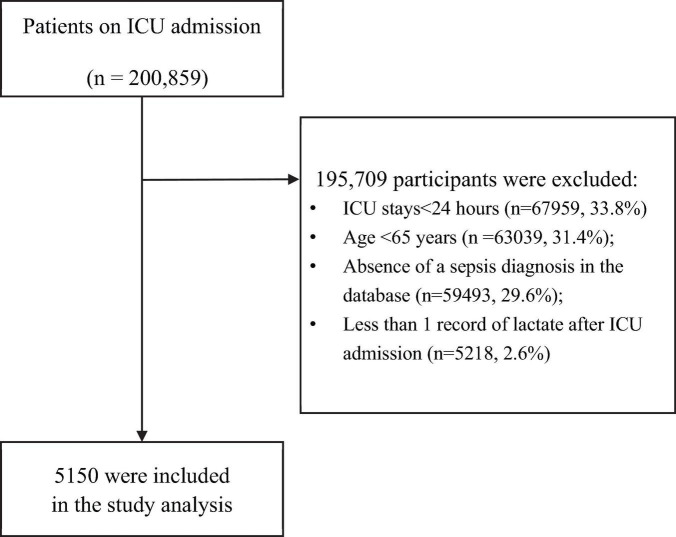
Flow chart of the study population. This flowchart illustrates the selection process of participants for the multicenter retrospective cohort study, showing the initial number of participants, exclusion criteria, and the final cohort of patients included in the analysis. ICU, intensive care unit.

This study was designed as a multicenter, retrospective cohort investigation. Initially, the dataset contained 200,859 ICU admissions. After applying the pre-specified exclusion criteria, 195,709 participants were excluded, resulting in a final cohort of 5,150 patients eligible for analysis. The exclusion criteria included: (1) ICU stays shorter than 24 h; (2) patients aged less than 65 years; (3) absence of a sepsis diagnosis; and (4) fewer than one serum lactate measurement recorded within 24 h post-ICU admission. Similar exclusion criteria have been widely adopted in numerous relevant studies, demonstrating the generalizability of this approach in clinical research (19, 20). A detailed flow diagram outlining the study selection process is shown in Figure 1.

### Data variables and covariates

The eICU-CRD provides a comprehensive array of data, including demographic details, physiological parameters recorded via bedside monitors, diagnoses coded according to the International Classification of Diseases, 9th Edition, Clinical Modification (ICD-9CM), and laboratory test results from routine clinical care. For this study, data were collected from the first 24 h following ICU admission. The primary exposure variable was the first serum lactate measurement recorded within 24 h of ICU admission, expressed in millimoles per liter (mmol/L). Several covariates were selected based on their clinical relevance and support from the literature, including: age (recorded at the time of admission), sex (male or female), and the presence of chronic conditions such as chronic obstructive pulmonary disease (COPD), chronic heart failure (CHF), acute myocardial infarction (AMI), and diabetes mellitus (DM). These conditions were categorized as either present or absent. Other variables included heart rate (HR), body temperature, hemoglobin concentration, potassium, red blood cells (RBC), sodium, white blood cells (WBC), bicarbonate levels, red cell distribution width (RDW), blood urea nitrogen (BUN), and the ICU unit type. Disease severity was quantified using the Acute Physiology and Chronic Health Evaluation (APACHE) score and the Sequential Organ Failure Assessment (SOFA) score, with higher scores reflecting greater severity. The primary outcome of interest was 28 days mortality, defined as death within 28 days of ICU admission.

### Statistical analysis

Descriptive statistics were employed to summarize the baseline characteristics of the study population. Continuous variables were expressed as means with standard deviations (SD) for normally distributed data or medians with interquartile ranges (IQR) for non-normally distributed data. Categorical variables were presented as frequencies and percentages. To compare variables across lactate level quartiles, one-way analysis of variance (ANOVA) was used for normally distributed continuous variables, the Kruskal-Wallis H test for skewed continuous variables, and the chi-squared test for categorical variables. A generalized additive model (GAM) was performed to explore the dose-response relationship between serum lactate levels and 28 days mortality. Univariate and multivariate binary logistic regression models were employed to assess the association between serum lactate levels and 28 days mortality. To adjust for potential confounders, two adjustment models were implemented, incorporating covariates such as age, sex, COPD, CHF, AMI, DM, HR, temperature, hemoglobin, potassium, RBC, sodium, WBC, bicarbonate, RDW, BUN, APACHE IV score, SOFA score, and ICU unit type. Subgroup analyses were conducted for several clinical variables, including HR, temperature, potassium, WBC, bicarbonate, RDW, BUN, APACHE IV score, and SOFA score, to further investigate the impact of these variables on the association between lactate levels and mortality. Results were presented as odds ratios (ORs) with corresponding 95% confidence intervals (CIs). To assess the threshold effect of lactate on 28 days mortality, a two-piece-wise linear regression model was applied. The turning point for lactate was identified through “exploratory” analyses, wherein the turning point was varied within a predetermined range to identify the value that maximized the model’s likelihood. Additionally, a log-likelihood ratio test was performed to compare the fit of the one-line linear regression model with the two-piecewise linear model. The bootstrap resampling method was employed to calculate the 95% CI for the turning point (21), as outlined in the previous analysis (22). To ascertain the robustness of the results, sensitivity analyses were conducted. All statistical analyses were carried out using R software version 4.0.0^1^ and Empower Stats (X&Y Solutions, Inc., Boston, MA). A two-sided *p*-value of less than 0.05 was considered statistically significant.

## Results

### Demographic and clinical characteristics

Data from 5,150 patients were analyzed. The median age was 76 years (IQR 70–83 years), 2,495 patients were males (48.45%). No significant difference in age was noted among the groups (*P* = 0.085), while a significant difference in gender distribution was observed (*P* = 0.020). HR increased significantly with higher lactate levels (*P* < 0.001), and the group Q4 recorded the lowest temperature (*P* < 0.001). Significant differences were also noted in other covariates, including hemoglobin, potassium, RBC, WBC, bicarbonate, RDW, BUN, APACHE IV score, and SOFA score (*P* < 0.001). The detailed baseline characteristics and associations between lactate and clinical variables are summarized in Table 1.

**TABLE 1:** Baseline characteristics of participants (*N* = 5,150).

Lactate (mmol/L) (quartiles)					
**Characteristics**	**Q1 < 1.5 *n* = 1,274**	**Q2 1.5–2.0 *n* = 1,238**	**Q3 2.1–3.27 *n* = 1,350**	**Q4 > 3.27 *n* = 1,288**	***P*-value**
Age (years)	76.37 ± 7.52	76.78 ± 7.58	77.12 ± 7.67	76.92 ± 7.73	0.085
Gender, *n* (%)					0.020
Male	662 (51.96)	576 (46.53)	630 (46.70)	627 (48.68)	–
Female	612 (48.04)	662 (53.47)	719 (53.30)	661 (51.32)	–
COPD, *n* (%)					0.022
No	1,144 (89.80)	1,118 (90.31)	1,217 (90.15)	1,197 (92.93)	–
Yes	130 (10.20)	120 (9.69)	133 (9.85)	91 (7.07)	–
CHF, *n* (%)					0.013
No	1,150 (90.27)	1,099 (88.77)	1,202 (89.04)	1188 (92.24)	–
Yes	124 (9.73)	139 (11.23)	148 (10.96)	100 (7.76)	–
AMI, *n* (%)					
No	1,224 (96.08)	1,173 (94.75)	1,282 (94.96)	1,228 (95.34)	–
Yes	50 (3.92)	65 (5.25)	68 (5.04)	60 (4.66)	–
DM, *n* (%)					0.725
No	1,100 (86.34)	1,066 (86.11)	1,150 (85.19)	1,116 (86.65)	–
Yes	174 (13.66)	172 (13.89)	200 (14.81)	172 (13.35)	–
HR (beats/min)	107.26 ± 28.52	111.91 ± 28.51	114.05 ± 28.35	117.09 ± 28.79	< 0.001
Temperature (°C)	36.56 ± 1.10	36.54 ± 1.30	36.56 ± 1.13	36.38 ± 1.45	< 0.001
Hemoglobin (g/dL)	10.08 ± 1.94	10.49 ± 2.09	10.56 ± 2.17	10.57 ± 2.28	< 0.001
Potassium (mmol/L)	4.03 ± 0.67	4.08 ± 0.74	4.13 ± 0.74	4.27 ± 0.87	< 0.001
RBC (M/mcl)	3.40 ± 0.64	3.55 ± 0.70	3.56 ± 0.75	3.56 ± 0.78	< 0.001
Sodium (mmol/L)	138.56 ± 6.00	139.15 ± 6.48	139.04 ± 6.41	139.09 ± 6.50	0.082
WBC (× 109/L)	13.40 (9.10–18.72)	14.30 (9.60–21.05)	15.20 (10.00–22.10)	15.47 (9.70–22.50)	< 0.001
Bicarbonate (mmol/L)	23.12 ± 5.11	22.67 ± 4.87	22.05 ± 4.94	19.00 ± 4.95	< 0.001
RDW (%)	15.93 ± 2.33	16.07 ± 2.50	16.14 ± 2.55	16.40 ± 2.73	< 0.001
BUN (mg/dL)	29.00 (19.00–47.00)	31.00 (21.00–49.00)	33.00 (22.00–49.00)	34.00 (24.00–51.00)	< 0.001
Apache IV score	71.53 ± 20.05	76.18 ± 22.74	79.36 ± 23.24	90.89 ± 27.85	< 0.001
SOFA score	4.00 (2.00–6.00)	4.00 (2.00–6.00)	5.00 (2.00–7.00)	6.00 (4.00–8.00)	< 0.001
Unittype					0.571
Medical surgical ICU	856 (67.19)	876 (70.76)	901 (66.74)	865 (67.16)	–
Neurological ICU	167 (13.11)	129 (10.42)	187 (13.85)	171 (13.28)	–
Coronary care					–
Unit/cardiot horacic ICU	86 (6.75)	80 (6.46)	80 (5.93)	75 (5.82)	–
Cardiothorac ic ICU	85 (6.67)	75 (6.06)	97 (7.19)	82 (6.37)	–
Medical ICU	30 (2.35)	31 (2.50)	33 (2.44)	42 (3.26)	–
Surgical ICU	27 (2.12)	17 (1.37)	18 (1.33)	23 (1.79)	–
Cardiac surgery ICU	7 (0.55)	8 (0.65)	9 (0.67)	9 (0.70)	–
Cardiac ICU	16 (1.26)	22 (1.78)	25 (1.85)	21 (1.63)	–
Time ICU 28 days	2.87 (1.85–4.88)	2.92 (1.84–5.13)	2.87 (1.84–5.01)	3.03 (1.81–5.50)	0.291
Mortality					< 0.001
No	1,177 (92.39)	1,127 (91.03)	1,176 (87.11)	959 (74.46)	–
Yes	97 (7.61)	111 (8.97)	174 (12.89)	329 (25.54)	–

Results in table: Mean (SD) Median (Q1−Q3)/*n* (%). Among the 5,150 patients, the amount of missing values for the covariates were 1 (0.02%) for gender; 127 (2.47%) for HR; 357 (6.93%) for temperature; 175 (3.40%) for hemoglobin; 96 (1.86%) for potassium; 201 (3.90%) for RBC; 107 (2.08%) for sodium; 191 (3.71%) for WBC; 326 (6.33%) for Bicarbonate; 482 (9.36%) for RDW; 116 (2.25%) for BUN; 607 (11.79%) for APACH IV score; 2 (0.04%) for SOFA score. COPD, chronic obstructive pulmonary disease; CHF, chronic heart failure; AMI, acute myocardial infarction; DM, diabetes mellitus; HR, heart rate; RBC, red blood cells; WBC, white blood cells; RDW, red cell distribution width; BUN, blood urea nitrogen; APACHE, acute physiology and chronic health evaluation; SOFA, sequential organ failure assessment; ICU, intensive care unit.

### 28 days mortality rates by lactate quartiles

The 28 days ICU mortality rate was 13.8% (711/5,150) with a 95% CI of 12.86–14.75 in our cohort. Mortality increased significantly with rising lactate levels, from 7.61% in the group Q1 to 25.54% in group Q4 (*P* < 0.001) (Table 1). The lower mortality compared to previous literature may be attributable to discrepancies in care practice and the severity of the disease.

### Univariate analysis for 28 days mortality

For each 1 mmol/L increase in lactate, mortality risk increased by 27% (OR = 1.27, 95% CI: 1.23–1.31, *P* < 0.0001). Quartile analysis revealed that patients in group Q4 (> 3.27 mmol/L) faced a significantly higher mortality risk (OR = 4.16, 95% CI: 3.27–5.30, *P* < 0.0001). Additionally, potassium (OR = 1.34, 95% CI: 1.21–1.47, *P* < 0.0001), RDW (OR = 1.12, 95% CI: 1.09–1.16, *P* < 0.0001), and SOFA score (OR = 1.24, 95% CI: 1.21–1.28, *P* < 0.0001) were significantly associated with increased mortality (Table 2).

**TABLE 2:** Univariate analysis for 28 days mortality in critically ill patients.

Exposure	Statistics	OR (95% CI)	*P*-value
Lactate (mmol/L)	2.10 (1.50–3.27)	1.27 (1.23, 1.31)	< 0.0001
**Lactate quartiles (mmol/L)**			
Q1 (< 1.5)	1,274 (24.74)	Reference	–
Q2 (1.5–2.0)	1,238 (24.04)	1.20 (0.90, 1.59)	0.2193
Q3 (2.1–3.27)	1,350 (26.21)	1.80 (1.38, 2.33)	< 0.0001
Q4 (> 3.27)	1,288 (25.01)	4.16 (3.27, 5.30)	< 0.0001
Age (years)	76.80 ± 7.63	0.99 (0.98, 1.00)	0.1846
**Gender, *n* (%)**			
Male	2,495 (48.46)	Reference	–
Female	2,654 (51.54)	1.01 (0.86, 1.18)	0.9021
**COPD, *n* (%)**			
No	4,676 (90.80)	Reference	–
Yes	474 (9.20)	0.88 (0.66, 1.17)	0.3685
**CHF, *n* (%)**			
No	4,639 (90.08)	Reference	–
Yes	511 (9.92)	1.20 (0.93, 1.54)	0.1584
**AMI, *n* (%)**			
No	4907 (95.28)	Reference	–
Yes	243 (4.72)	1.28 (0.91, 1.81)	0.1567
**DM, *n* (%)**			
No	4,432 (86.06)	Reference	–
Yes	718 (13.94)	0.79 (0.62, 1.01)	0.0605
HR (beats/min)	112.63 ± 28.76	1.01 (1.01, 1.01)	< 0.0001
Temperature (°C)	36.51 ± 1.25	0.84 (0.79, 0.90)	< 0.0001
Hemoglobin (g/dL)	10.43 ± 2.14	0.97 (0.93, 1.00)	0.0715
Potassium (mmol/L)	4.13 ± 0.76	1.34 (1.21, 1.47)	< 0.0001
RBC (M/mcl)	3.52 ± 0.72	0.91 (0.81, 1.02)	0.0946
Sodium (mmol/L)	138.96 ± 6.35	1.00 (0.99, 1.01)	0.8846
WBC (× 109/L)	14.40 (9.60–21.18)	1.01 (1.00, 1.01)	0.0149
Bicarbonate (mmol/L)	21.69 ± 5.22	0.93 (0.91, 0.95)	< 0.0001
RDW (%)	16.14 ± 2.54	1.12 (1.09, 1.16)	< 0.0001
BUN (mg/dL)	16.14 ± 2.54	1.01 (1.01, 1.01)	< 0.0001
Apache IV score	79.56 ± 24.71	1.03 (1.03, 1.04)	< 0.0001
SOFA score	5.00 (2.00–7.00)	1.24 (1.21, 1.28)	< 0.0001

Results in table: Mean (SD) Median (Q1−Q3)/*n* (%). COPD, chronic obstructive pulmonary disease; CHF, chronic heart failure; AMI, acute myocardial infarction; DM, diabetes mellitus; HR, heart rate; RBC, red blood cells; WBC, white blood cells; RDW, red cell distribution width; BUN, blood urea nitrogen; APACHE, acute physiology and chronic health evaluation; SOFA, sequential organ failure assessment.

### Relationship between lactate and 28 days mortality

In the unadjusted model, a 1 mmol/L increase in lactate was related to a 27% increase in mortality risk (OR = 1.27, 95% CI: 1.23–1.31, *P* < 0.0001). In Model II, after adjusting for covariates, the OR was reduced to 1.16 (95% CI: 1.11–1.21, *P* < 0.0001), demonstrating that high lactate levels are independently associated with an increased mortality risk. Quartile analysis showed that patients in group Q4 (> 3.27 mmol/L) faced a notably higher mortality risk across all models, especially in Model II (OR = 2.27, 95% CI: 1.66–3.10, *P* < 0.0001) (Table 3). Supplementary analysis yielded similar results, even when considering the impact of missing data (Supplementary Table 1).

**TABLE 3:** Relationship between lactate and 28°days mortality.

Outcome	Crude model		Model I		Model II	
	**OR (95% CI)**	***P*-value**	**OR (95% CI)**	***P*-value**	**OR (95% CI)**	***P*-value**
Lactate (mmol/L)	1.27 (1.23, 1.31)	< 0.0001	1.27 (1.23, 1.31)	< 0.0001	1.16 (1.11, 1.21)	< 0.0001
**Lactate quartiles (mmol/L)**						
Q1 (< 1.5)	Reference	–	Reference	–	Reference	–
Q2 (1.5– 2.0)	1.20 (0.90, 1.59)	0.2193	1.20 (0.90, 1.59)	0.2107	0.92 (0.65, 1.30)	0.6355
Q3 (2.1–3.27)	1.80 (1.38, 2.33)	< 0.0001	1.81 (1.39, 2.35)	< 0.0001	1.35 (0.98, 1.86)	0.0623
Q4 (> 3.27)	4.16 (3.27, 5.30)	< 0.0001	4.19 (3.29, 5.33)	< 0.0001	2.27 (1.66, 3.10)	< 0.0001

Model I adjusted for age and gender. Model II adjusted for age, gender, chronic obstructive pulmonary disease (COPD), chronic heart failure (CHF), acute myocardial infarction (AMI), diabetes mellitus (DM), heart rate (HR), temperature, hemoglobin, potassium, red blood cells (RBC), sodium, white blood cells (WBC), bicarbonate, red cell distribution width (RDW), blood urea nitrogen (BUN), acute physiology and chronic health evaluation (APACHE) IV score, sequential organ failure assessment (SOFA) score, unit type. OR, odds ratio; CI, confidence interval.

### Effect size of lactate on 28 days mortality in prespecified and exploratory subgroups

**FIGURE 2:**
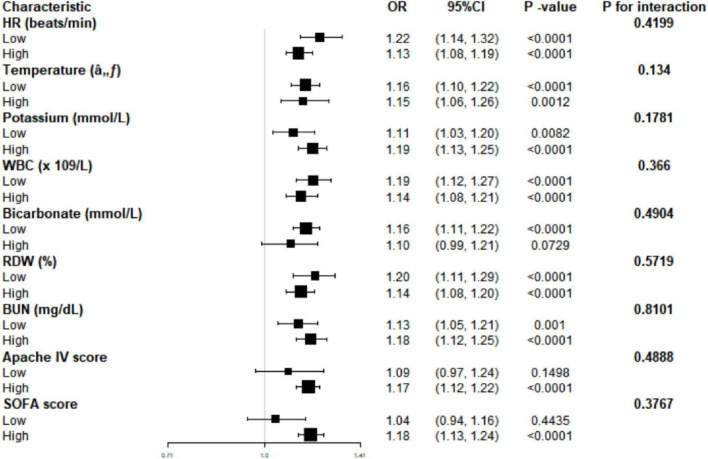
Effect size of lactate on 28 days mortality in prespecified and exploratory subgroups. HR, heart rate; WBC, white blood cells; RDW, red cell distribution width; BUN, blood urea nitrogen; APACHE, acute physiology and chronic health evaluation; SOFA, sequential organ failure Assessment; OR, odds ratio; CI, confidence interval.

Stratified analysis according to HR, temperature, potassium, WBC, bicarbonate, RDW, BUN, Apache IV, and SOFA scores confirmed lactate as a significant predictor of 28 days mortality, particularly in subgroups with low HR (OR = 1.22, 95% CI: 1.14–1.32, *P* < 0.0001), high potassium (OR = 1.19, 95% CI: 1.13–1.25, *P* < 0.0001), low WBC (OR = 1.19, 95% CI: 1.12–1.27, *P* < 0.0001), and low RDW (OR = 1.20, 95% CI: 1.11–1.29, *P* < 0.0001). Interaction analysis indicated that covariates did not significantly alter the correlation between lactate and mortality, suggesting lactate’s independent predictive value for mortality (Figure 2). These findings underscore the robust detrimental effect of lactate on 28 days mortality across various patient characteristics and clinical conditions.

## Identification of non-linear relationship

### The threshold effect of lactate level on 28 days ICU mortality in elderly patients with sepsis

**FIGURE 3:**
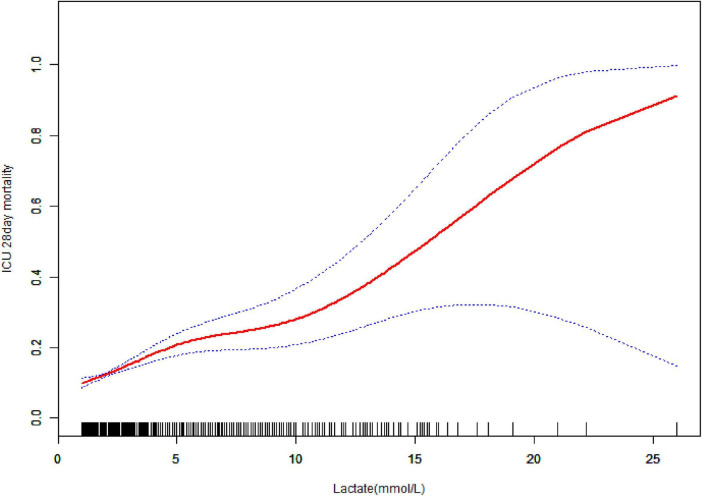
Associations between the lactate and 28 days mortality in elderly patients with sepsis. A threshold, non-linear association between the lactate and 28 days mortality was found in a generalized additive model (GAM). Solid rad line represents the smooth curve fit between variables. Blue bands represent the 95% of confidence interval from the fit. Adjusted for age, gender, chronic obstructive pulmonary disease (COPD), chronic heart failure (CHF), acute myocardial infarction (AMI), diabetes mellitus (DM), heart rate, temperature, hemoglobin, potassium, red blood cells (RBC), sodium, white blood cells (WBC), bicarbonate, red cell distribution width (RDW), blood urea nitrogen (BUN), acute physiology and chronic health evaluation (APACHE) IV score, sequential organ failure assessment (SOFA) score, unit type.

A non-linear dose–response relationship was represented between lactate and mortality (Figure 3 and Table 4). A positive correlation was identified when lactate was < 3.7 mmol/L, with an adjusted OR of 1.33 (95% CI: 1.17–1.51, *P* < 0.0001) for every 1 mmol/L increase. For lactate ≥ 3.7 mmol/L, mortality increased with an adjusted OR of 1.11 (95% CI: 1.05–1.18, *P* = 0.0003) per 1 mmol/L increase (Table 4). Using a generalized additive model, a non-linear association was revealed between lactate and 28 days mortality (Table 4). A comparison between the linear regression model and a two-piece-wise linear regression model was conducted, and the log-likelihood ratio test yielded a *P*-value of less than 0.026. These results present that the two-piece-wise linear regression model is more suitable for modeling the data. Supplementary analysis yielded similar results, even after considering the impact of missing data (Supplementary Figure 1 and Supplementary Table 2).

**TABLE 4:** Threshold effect analysis of the lactate and 28 days mortality.

Models	OR (95% CI)	*P*-value
**Model I**		
One line effect	1.16 (1.11, 1.21)	< 0.0001
**Model II**		
Turning point (K)	3.7	–
Lactate < K	1.33 (1.17, 1.51)	< 0.0001
Lactate ≥ K	1.11 (1.05, 1.18)	0.0003
*P*-value for LRT test*	–	0.026

Data were presented as OR (95% CI), *P*-value; Model I, linear analysis; Model II, non-linear analysis. Adjusted for age, gender, chronic obstructive pulmonary disease (COPD), chronic heart failure (CHF), acute myocardial infarction (AMI), diabetes mellitus (DM), heart rate, temperature, hemoglobin, potassium, red blood cells (RBC), sodium, white blood cells (WBC), bicarbonate, red cell distribution width (RDW), blood urea nitrogen (BUN), acute physiology and chronic health evaluation (APACHE) IV score, sequential organ failure assessment (SOFA) score, unit type.

*P < 0.05 indicates that model II is significantly different from Model I. OR, odds ratio; CI, confidence interval; LRT, logarithm likelihood ratio test.

## The relationship between lactate and 28 days ICU mortality in sofa score categories

**FIGURE 4:**
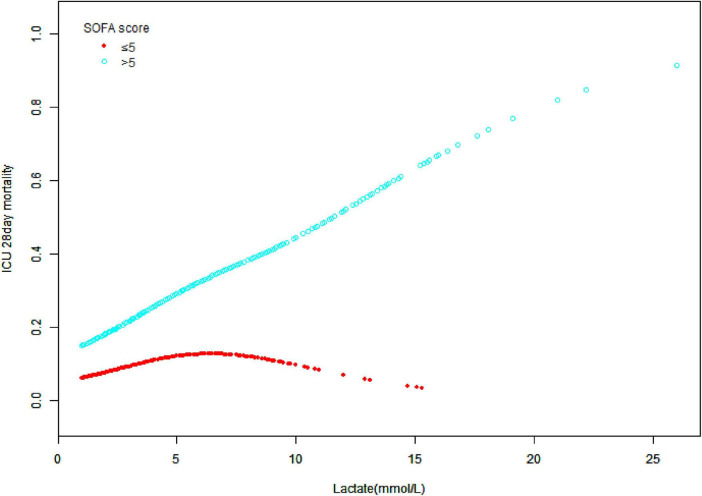
Associations between lactate and 28 days intensive care unit (ICU) mortality across different sequential organ failure assessment (SOFA) score categories. A statistical analysis of the data with lactate levels as the exposure variable, ICU 28 days mortality as the outcome variable, and SOFA score as the stratification factor.

Figure 4 illustrates the correlation between lactate and 28 days ICU mortality across different SOFA score categories. This study performed statistical analysis using lactate levels as the exposure variable, 28 days ICU mortality as the outcome variable, and SOFA score as the stratification factor. In the SOFA score ≤ 5 group, mortality was low but increased gradually with rising lactate levels. Conversely, in the SOFA score > 5 group, mortality rose significantly, especially when lactate levels exceeded 5 mmol/L. Patients with higher SOFA scores exhibited a greater mortality risk as lactate levels increased. The distinct separation of the curves emphasizes the critical role of lactate levels and SOFA scores in assessing patient prognosis. Supplementary analysis yielded similar results, even after considering the impact of missing data (Supplementary Figure 2).

The sensitivity analysis results aligned consistently with those of the main analysis. In the supplementary analysis, we further excluded patients who died within 24 h, and the findings remained largely consistent with the main results (Supplementary Figures 3, 4 and Supplementary Table 3). Moreover, time-dependent analyses were conducted, yielding outcomes that were generally consistent with the main findings (Supplementary Figures 5, 6 and Supplementary Table 4).

### Discussion

In this study, a clear positive correlation was identified between lactate levels and 28 days mortality in elder patients with sepsis across different SOFA score groups. A non-linear relationship with a threshold effect was noted: below 3.7 mmol/L, each increment in lactate was related to a significant increase in mortality risk (OR = 1.33, 95% CI: 1.17–1.51, *P* < 0.0001). Above 3.7 mmol/L, the increase in mortality risk was less pronounced (OR = 1.11, 95% CI: 1.05–1.18, *P* = 0.0003). Stratification by SOFA score revealed that patients with higher scores faced a heightened mortality risk as lactate levels increased. To our knowledge, this is the first research to extensively explore the relationship between lactate levels and 28 days mortality in elderly sepsis patients across different SOFA score groups.

Elevated lactate levels were positively associated with mortality in patients with sepsis, as supported by several studies that highlight the significance of lactate as a prognostic marker in this cohort. One investigation found that a baseline blood lactate level exceeding 2.5 mmol/L was associated with a notably higher mortality rate (16.9% vs. 5.8%, *P* < 0.001) among patients with severe sepsis and septic shock (23). Additionally, research indicated that initial lactate levels exceeding 36 mg/dL were significantly associated with increased mortality in pediatric sepsis (adjusted OR: 3.26; 95% CI: 1.16–9.16) (24). Moreover, another study investigated the prognostic significance of lactate levels in patients with septic shock, revealing that lactate levels of ≥ 2 mmol/L exhibited the highest sensitivity of 85.3% (25). In summary, while variations in lactate thresholds exist across different studies, the collective evidence indicate that increased lactate levels are significantly correlated with the 28 days mortality in elderly septic patients. Furthermore, a study involving 1,371 patients with septic shock identified the normalized lactate load as a risk factor for mortality, further corroborating the relationship between lactate levels and mortality, with higher levels linked to increased 28 days mortality (26). However, this study differs from previous research as it encompasses a larger sample size, and specifically focuses on elderly patients with sepsis in the ICU.

Another study involving 4,199 elderly patients with sepsis demonstrated that for each 1 mmol/L increase in lactate, the OR for 28 days mortality was 1.23 (95% CI: 1.18–1.28, *P* < 0.0001) (10). The study also identified a non-linear positive correlation between lactate and 28 days mortality, with a turning point at 5.7 mmol/l. Below this level, a 32% increase in the risk of 28 days mortality was observed for each 1 mmol/L increase in lactate (OR: 1.32, 95%CI: 1.25–1.38, *P* < 0.0001). This significant positive relationship persisted at lactate levels above 5.7 mmol/L (OR: 1.10, 95% CI: 1.04–1.18, *P* = 0.0019). In our study, the 28 days ICU mortality was lower at 13.8% and the turning point was significantly different (3.7 mmol/l compared to 5.7 mmol/l). Discrepancies in outcomes may be attributed to the larger sample size of this study, use of data from different databases across different countries, and inclusion of different covariates. Moreover, this study employed the SOFA score as a stratification factor for the analysis, enabling a more detailed examination of the relationship between lactate and mortality in different SOFA score groups.

The Surviving Sepsis Campaign underscores the pivotal role of expeditious identification of elevated lactate levels in facilitating timely intervention and enhancing prognoses, particularly in high-risk patients (27). The non-linear relationship observed between lactate levels and mortality in this study further supports the clinical application of lactate as a prognostic indicator. Furthermore, this study suggests the potential need to establish more stringent intervention thresholds in future clinical practice. This aligns with the guidelines’ principle of “timely identification and management of elevated lactate levels.”

A study with inconsistent results reported that elderly patients with sepsis had significantly lower lactate levels, approximately 1 mmol/L lower (6.6 vs. 5.5, *P* < 0.01), in the non-survival group compared to non-elderly patients (6). Discrepancies in findings might be attributed to variations in study populations, sample sizes, selection of covariates, and data analysis strategies. The earlier study focused on adult Asian patients in the emergency department who received intravenous antibiotic treatment. In contrast, our research targeted elderly patients in the ICU, including a more diverse racial background and a larger sample size. Distinct populations may exhibit unique physiological states and mortality risk factors, and racial differences could also be significant (6). Furthermore, the covariates in the two studies differed. Previous research did not include factors relevant to septic patients, such as the SOFA score, and was primarily concerned with major sources of infection. Conversely, our study included the SOFA score, the Apache IV score, and multiple laboratory parameters, which are particularly important for elderly patients with sepsis in the ICU setting. Lastly, the statistical methods applied varied. The previous study used receiver operating characteristic (ROC) curve analysis to identify optimal clinical cut-off points, while our research conducted a threshold effect analysis to assess the non-linear impact of lactate levels on mortality and to identify its turning point. These methodological differences may affect the interpretation of results, especially concerning non-linear relationships and threshold effects (28).

The elevation of lactate levels in sepsis is primarily due to several mechanisms. Microcirculation disorders and tissue hypoxia induce cells to produce lactate through the anaerobic metabolic pathway to meet energy demands (29). Additionally, elevated levels of stress hormones (such as adrenaline and cortisol) enhance glycogenolysis and lactate production (30). Bacterial infections may also release metabolites, impacting the metabolic status of the host (31). Moreover, local underperfusion of the liver reduces lactate clearance efficiency, leading to increased lactate levels in the blood (32, 33). The interaction of these mechanisms heightens the mortality risk associated with elevated lactate levels in elderly patients with sepsis. The stratification of SOFA scores significantly impacts the prognostic value of lactate, with this relationship potentially being related to organ function status, inflammatory burden, and microcirculation (34, 35). In patients exhibiting low SOFA scores (≤ 5), despite elevated lactate levels, the maintenance of adequate organ function results in a slower increase in mortality, indicating strong metabolic adaptation and lactate clearance capabilities. Conversely, in patients with high SOFA scores (> 5), organ dysfunction leads to decreased lactate metabolism and clearance. Additionally, reduced hepatic perfusion and increased inflammatory burden further exacerbate metabolic abnormalities, thereby elevating the mortality risk in patients with high lactate levels.

This study fills a notable gap in the literature by highlighting the role of lactate in predicting 28 days mortality risk among elderly patients with sepsis across different SOFA score groups. Results demonstrate that lactate levels are independently associated with 28 days mortality risk in these patients, even after adjusting for established factors. Stratification by SOFA score showed that patients with higher scores faced elevated mortality risks as lactate levels increased, suggesting varying impacts of lactate on mortality risk depending on SOFA scores. One study found that the SOFA score could effectively predict 28 days mortality in elderly sepsis patients, revealing significantly higher mortality rates for those with a SOFA score of 2 compared to those with lower scores (36). It is recommended that clinicians consider lactate thresholds as early warning indicators for identifying high-risk patients. For patients with a SOFA score > 5 and lactate > 5 mmol/L, priority should be given to ICU admission, while patients with an initial lactate ≥ 3.7 mmol/L require close observation. Furthermore, it is recommended that lactate thresholds be incorporated into existing early warning scores, particularly for the elderly sepsis patients. In conclusion, the present study highlights the intricate relationship between lactate levels and mortality risk in elderly sepsis patients, emphasizing the significance of personalized lactate evaluation and intervention based on SOFA scores in clinical management.

Moreover, future research, encompassing prospective studies and randomized controlled trials, is essential to explore the underlying mechanisms of the correlation between lactate and mortality. Such research would provide more substantial evidence for clinical practice. A principal strength of this study is its utilization of multicenter data, which ensures a nationally representative cohort of elderly sepsis patients and enhances the generalizability of the findings. The study employed OR and 95% CI, adjusted for multiple covariates, and conducted stratified analyses to ascertain the robustness and variability of the results. Besides, the threshold effect of lactate on 28 days mortality was examined applying a two-piece-wise linear regression model, identifying notable turning points in lactate levels. The introduction of stratifying factors allowed for the identification of significant differences between groups. These findings suggest that future studies should include a broader range of stratifying factors to fully understand their potential impact on outcomes.

Nevertheless, the present study is subject to limitations due to potential unmeasured confounding factors, including fluid resuscitation, the application of vasopressors, and variations in ICU care variations. Despite the challenges associated with quantification, their overall impact is relatively limited and insufficient to undermine the validity of the study’s conclusion (37, 38). Another limitation involves missing data for certain variables; to tackle this and minimize bias, the median imputation were applied. Furthermore, the study focused exclusively on 28 days mortality, without evaluating long-term outcomes. Additionally, this study included only the initial lactate measurement within 24 h of ICU admission, which does not reflect temporal variation in lactate levels or their impact on prognosis. Besides, the exclusion of patients with ICU stays under 24 h may result in bias, including the potential underestimation of mortality among patients with early, severe hyperlactatemia. Despite this, it is the contention of the present study that the conclusions drawn remain applicable to the target population. The data in the eICU database were collected from 2014 to 2015. Significant advancements in the management of sepsis have occurred in recent years, including the Sepsis-3 definition and updated treatment guidelines (2, 27). However, these historical data provide important insights into the characteristics and management of sepsis. As an observational study, the relationship between lactate and 28 days mortality is correlational rather than causal. Furthermore, as the inclusion criteria were restricted to elderly patients with sepsis, the findings may not be generalizable to younger sepsis patients. Moreover, this is a retrospective study according to a US public database, and the conclusions may not apply to non-United States healthcare settings, thereby limiting the generalizability of the findings.

### Conclusion

Data from the eICU-CRD were utilized to identify 5,150 elderly patients with sepsis. This study reveals a non-linear positive relationship between lactate levels and 28 days mortality among elderly sepsis patients. Furthermore, stratification by SOFA score demonstrated that patients with higher scores exhibited a heightened risk of mortality as lactate levels increased.

## Data Availability

The datasets presented in this study can be found in online repositories. The names of the repository/repositories and accession number(s) can be found below: https://physionet.org/content/eicu-crd/2.0/.

## Footnotes

1
http://www.r-project.org

## References

[1] Angus D Linde-Zwirble W Lidicker J Clermont G Carcillo J Pinsky M. Epidemiology of severe sepsis in the United States: Analysis of incidence, outcome, and associated costs of care. *Crit Care Med.* (2001) 29:1303–10. 10.1097/00003246-200107000-00002 11445675

[2] Shankar-Hari M Phillips G Levy M Seymour C Liu V Deutschman C Developing a new definition and assessing new clinical criteria for septic shock: For the third international consensus definitions for sepsis and septic shock (Sepsis-3). *JAMA.* (2016) 315:775–87. 10.1001/jama.2016.0289 26903336 PMC4910392

[3] Rudd K Johnson S Agesa K Shackelford K Tsoi D Kievlan D Global, regional, and national sepsis incidence and mortality, 1990-2017: Analysis for the Global burden of disease study. *Lancet.* (2020) 395:200–11. 10.1016/S0140-6736(19)32989-7 31954465 PMC6970225

[4] Hernández-Quiles R Merino-Lucas E Boix V Fernández-Gil A Rodríguez-Díaz J Gimeno A Bacteraemia and quick sepsis related organ failure assessment (qSOFA) are independent risk factors for long-term mortality in very elderly patients with suspected infection: Retrospective cohort study. *BMC Infect Dis.* (2022) 22:248. 10.1186/s12879-022-07242-4 35279079 PMC8918285

[5] Devia Jaramillo G Ibáñez Pinilla M. Quick sequential organ failure assessment, sequential organ failure assessment, and procalcitonin for early diagnosis and prediction of death in elderly patients with suspicion of sepsis in the emergency department, based on sepsis-3 definition. *Gerontology.* (2022) 68:171–80. 10.1159/000515851 33951628

[6] Cheng H Chen F Change M Kung C Cheng C Tsai T Difference between elderly and non-elderly patients in using serum lactate level to predict mortality caused by sepsis in the emergency department. *Medicine (Baltimore).* (2018) 97:e0209. 10.1097/MD.0000000000010209 29595662 PMC5895436

[7] Evans L Rhodes A Alhazzani W Antonelli M Coopersmith C French C Surviving sepsis campaign: International guidelines for management of sepsis and septic shock 2021. *Crit Care Med.* (2021) 49:e1063–143. 10.1097/CCM.0000000000005337 34605781

[8] Xie Y Hu H Liu M Zhou T Cheng X Huang W The role and mechanism of histone lactylation in health and diseases. *Front Genet.* (2022) 13:949252. 10.3389/fgene.2022.949252 36081996 PMC9445422

[9] Vink E Bakker J. Practical use of lactate levels in the intensive care. *J Intensive Care Med.* (2018) 33:159–65. 10.1177/0885066617708563 28486864

[10] He L Yang D Ding Q Su Y Ding N. Association Between lactate and 28-day mortality in elderly patients with sepsis: Results from MIMIC-IV database. *Infect Dis Ther.* (2023) 12:459–72. 10.1007/s40121-022-00736-3 36520327 PMC9925625

[11] Burstein B Vallabhajosyula S Ternus B Barsness G Kashani K Jentzer J. The prognostic value of lactate in cardiac intensive care unit patients with cardiac arrest and *shock*. *Shock.* (2021) 55:613–9. 10.1097/SHK.0000000000001582 32496423

[12] Tovoli F Ferri S Piscaglia F. Hepatocellular carcinoma in non alcoholic fatty liver disease. *Curr Pharm Des.* (2020) 26:3909–14. 10.2174/1381612826666200429093648 32348210

[13] Zeng Z Huang R Lin H Peng H Luo J Ding N. Serum lactate is an indicator for short-term and long-term mortality in patients with acute pancreatitis. *Dig Dis Sci.* (2024) 69:2223–34. 10.1007/s10620-024-08419-4 38594436

[14] Aksu A Gulen M Avci A Satar S. Adding lactate to SOFA and qSOFA scores predicts in-hospital mortality better in older patients in critical care. *Eur Geriatr Med.* (2019) 10:445–53. 10.1007/s41999-019-00179-z 34652794

[15] Xu H Xu Y He Y Lin X Suo Z Shu H Association between admission pan-immune-inflammation value and short-term mortality in septic patients: A retrospective cohort study. *Sci Rep.* (2024) 14:15205. 10.1038/s41598-024-66142-6 38956306 PMC11219806

[16] Xu Z Huang MA. dynamic nomogram for predicting 28-day mortality in septic shock: A Chinese retrospective cohort study. *PeerJ.* (2024) 12:e16723. 10.7717/peerj.16723 38282860 PMC10812607

[17] Chang L Chen X Lian C. The association between the non-HDL-cholesterol to HDL-cholesterol ratio and 28-day mortality in sepsis patients: A cohort study. *Sci Rep.* (2022) 12:3476. 10.1038/s41598-022-07459-y 35241749 PMC8894387

[18] Song J Yu T Yan Q Wu L Li S Wang LA. simple APACHE IV risk dynamic nomogram that incorporates early admitted lactate for the initial assessment of 28-day mortality in critically ill patients with acute myocardial infarction. *BMC Cardiovasc Disord.* (2022) 22:502. 10.1186/s12872-022-02960-8 36434509 PMC9700900

[19] Yu X Xin Q Hao Y Zhang J Ma T. An early warning model for predicting major adverse kidney events within 30 days in sepsis patients. *Front Med (Lausanne).* (2024) 10:1327036. 10.3389/fmed.2023.1327036 38469459 PMC10925638

[20] Cui K Mao Y Feng S Luo H Yang J Xu R Association between age and the 28-day all-cause mortality in tuberculosis complicated by sepsis in ICU patients: A retrospective cohort study [response to letter]. *Infect Drug Resist.* (2024) 17:2711–2. 10.2147/IDR.S484233 38974313 PMC11226857

[21] Lin L Chen C Yu X. [The analysis of threshold effect using Empower Stats software]. *Zhonghua Liu Xing Bing Xue Za Zhi.* (2013) 34:1139–41. 10.3760/cma.j.issn.0254-6450.2013.011.021 24517951

[22] Yu X Chen J Li Y Liu H Hou C Zeng Q Threshold effects of moderately excessive fluoride exposure on children’s health: A potential association between dental fluorosis and loss of excellent intelligence. *Environ Int.* (2018) 118:116–24. 10.1016/j.envint.2018.05.042 29870912

[23] Filho R Rocha L Corrêa T Pessoa C Colombo G Assuncao M. Blood lactate levels cutoff and mortality prediction in sepsis-time for a reappraisal? A retrospective cohort study. *Shock.* (2016) 46:480–5. 10.1097/SHK.0000000000000667 27380535 PMC5058781

[24] Scott H Brou L Deakyne S Kempe A Fairclough D Bajaj L. Association between early lactate levels and 30-day mortality in clinically suspected sepsis in children. *JAMA Pediatr.* (2017) 171:249–55. 10.1001/jamapediatrics.2016.3681 28068437

[25] Ryoo S Lee J Lee Y Lee J Lim K Huh J Lactate level versus lactate clearance for predicting mortality in patients with septic shock defined by sepsis-3. *Crit Care Med.* (2018) 46:e489–95. 10.1097/CCM.0000000000003030 29432347

[26] Chen H Gong S Yu R. Association between normalized lactate load and mortality in patients with septic shock: An analysis of the MIMIC-III database. *BMC Anesthesiol.* (2021) 21:16. 10.1186/s12871-021-01239-3 33435876 PMC7802303

[27] Zheng R Zhang Y Rong Z Huang W Fu X. [Surviving sepsis campaign: International guidelines for management of sepsis and septic shock 2021, interpretation and expectation]. *Zhonghua Wei Zhong Bing Ji Jiu Yi Xue.* (2021) 33:1159–64. 10.3760/cma.j.cn121430-20211009-01442 34955122

[28] Tu J Fang Q Zhao A Wu J. Discussion on the interpretation of the results and selection of the effect model in a meta-analysis. *Ann Palliat Med.* (2022) 11:1153–4. 10.21037/apm-21-3819 35272465

[29] Casserly B Phillips G Schorr C Dellinger R Townsend S Osborn T Lactate measurements in sepsis-induced tissue hypoperfusion: Results from the Surviving Sepsis Campaign database. *Crit Care Med.* (2015) 43:567–73. 10.1097/CCM.0000000000000742 25479113

[30] Vandewalle J Timmermans S Paakinaho V Vancraeynest L Dewyse L Vanderhaeghen T Combined glucocorticoid resistance and hyperlactatemia contributes to lethal shock in sepsis. *Cell Metab.* (2021) 33:1763–76.e5. 10.1016/j.cmet.2021.07.002 34302744

[31] Bulik-Sullivan E Roy S Elliott R Kassam Z Lichtman S Carroll I Intestinal microbial and metabolic alterations following successful fecal microbiota transplant for D-Lactic acidosis. *J Pediatr Gastroenterol Nutr.* (2018) 67:483–7. 10.1097/MPG.0000000000002043 29901551

[32] Tapia P Soto D Bruhn A Alegría L Jarufe N Luengo C Impairment of exogenous lactate clearance in experimental hyperdynamic septic shock is not related to total liver hypoperfusion. *Crit Care.* (2015) 19:188. 10.1186/s13054-015-0928-3 25898244 PMC4432956

[33] Lado-Abeal J Martinez-Sánchez N Cocho J Martín-Pastor M Castro-Piedras I Couce-Pico M Lipopolysaccharide (LPS)-induced septic shock causes profound changes in myocardial energy metabolites in pigs. *Metabolomics.* (2018) 14:131. 10.1007/s11306-018-1433-x 30830414

[34] Park H Lee J Oh D Park M Lim C Lee S Serial evaluation of the serum lactate level with the SOFA score to predict mortality in patients with sepsis. *Sci Rep.* (2023) 13:6351. 10.1038/s41598-023-33227-7 37072424 PMC10113181

[35] Liu F Jin F Zhang L Tang Y Wang J Zhang H Lactate combined with SOFA score for improving the predictive efficacy of SOFA score in patients with severe heatstroke. *Am J Emerg Med.* (2024) 78:163–9. 10.1016/j.ajem.2024.01.033 38295465

[36] Gaini S Relster M Pedersen C Johansen I. Prediction of 28-days mortality with sequential organ failure assessment (SOFA), quick SOFA (qSOFA) and systemic inflammatory response syndrome (SIRS) - A retrospective study of medical patients with acute infectious disease. *Int J Infect Dis.* (2019) 78:1–7. 10.1016/j.ijid.2018.09.020 30267939

[37] Chen H Zhao C Wei Y Jin J. Early lactate measurement is associated with better outcomes in septic patients with an elevated serum lactate level. *Crit Care.* (2019) 23:1–11. 10.1186/s13054-019-2625-0 31711512 PMC6849274

[38] Chen H Gong S Yu R. Increased normalized lactate load is associated with higher mortality in both sepsis and non-sepsis patients: An analysis of the MIMIC-IV database. *BMC Anesthesiol.* (2022) 22:79. 10.1186/s12871-022-01617-5 35337269 PMC8951714

