# Supplementary material for: Non-linear association between lactate and 28 days mortality in elderly patients with sepsis across different SOFA score groups: results from the eICU Collaborative Research Database
Source: Front Med (Lausanne). 2025 Jul 3;12:1605319. doi: 10.3389/fmed.2025.1605319 (PMC12267231; doi:10.3389/fmed.2025.1605319)
Supplement: Supplementary file 1 [file Table_1.docx]

**Supplementary File**

**Supplementary Table 1: Relationship between lactate and** **28-day mortality**

| Outcome | Crude Model | |  | Model Ⅰ | |  | Model Ⅱ | |
| --- | --- | --- | --- | --- | --- | --- | --- | --- |
|  | OR(95%CI) | P-value |  | OR(95%CI) | P-value |  | OR(95%CI) | P-value |
| Lactate (mmol/L) | 1.27(1.23, 1.31) | <0.0001 |  | 1.27(1.23, 1.31) | <0.0001 |  | 1.19 (1.16, 1.24) | <0.0001 |
| Lactate quartiles (mmol/L) |  |  |  |  |  |  |  |  |
| Q1 (<1.5) | Reference |  |  | Reference |  |  | Reference |  |
| Q2 (1.5-2.0) | 1.20(0.90, 1.59) | 0.2193 |  | 1.20(0.90, 1.59) | 0.2107 |  | 1.02 (0.76, 1.37) | 0.8804 |
| Q3 (2.1-3.27) | 1.80(1.38, 2.33) | <0.0001 |  | 1.81(1.39, 2.35) | <0.0001 |  | 1.48 (1.13, 1.94) | 0.0047 |
| Q4 (>3.27) | 4.16(3.27, 5.30) | <0.0001 |  | 4.19(3.29, 5.33) | <0.0001 |  | 2.77 (2.13, 3.60) | <0.0001 |

OR, odds ratio; CI, confidence interval. Model Ⅰadjusted for age and gender. Model Ⅱadjusted for age, gender, COPD, CHF, AMI, DM, heart rate, temperature, hemoglobin, potassium, RBC, sodium, WBC, bicarbonate, RDW, BUN, Apache IV score, SOFA score, unittype.

**Supplementary Table 2: Threshold effect analysis of the lactate and 28-day mortality**

| **Models** | **OR (95%CI)** | ***P*** value |
| --- | --- | --- |
| Model I | | |
| One line effect | 1.19 (1.15, 1.23) | <0.0001 |
| Model II | | |
| Turning point (K) | 3.4 | |
| Lactate < K | 1.44 (1.28, 1.62) | <0.0001 |
| Lactate ≥ K | 1.13 (1.09, 1.19) | <0.0001 |
| P value for LRT test* |  | <0.001 |
| Data were presented as OR (95% CI) P value; Model I, linear analysis; Model II, non-linear analysis. Adjusted for age, gender, COPD, CHF, AMI, DM, heart rate, temperature, hemoglobin, potassium, RBC, sodium, WBC, bicarbonate, RDW, BUN, Apache IV score, SOFA score, unittype. OR, odds ratio; CI, confidence interval; LRT, logarithm likelihood ratio test. * P<0.05 indicates that model II is significantly different from Model I. | | |

**Supplementary Table 3: Threshold effect analysis of the lactate and 28-day mortality**

| **Models** | **OR (95%CI)** | ***P*** value |
| --- | --- | --- |
| Model I | | |
| One line effect | 1.19 (1.16, 1.22) | <0.0001 |
| Model II | | |
| Turning point (K) | 3.88 | |
| Lactate < K | 1.44 (1.34, 1.55) | <0.0001 |
| Lactate ≥ K | 1.12 (1.08, 1.15) | <0.0001 |
| P value for LRT test* |  | <0.001 |
| Data were presented as OR (95% CI) P value; Model I, linear analysis; Model II, non-linear analysis. Adjusted for age, gender, COPD, CHF, AMI, DM, heart rate, temperature, hemoglobin, potassium, RBC, sodium, WBC, bicarbonate, RDW, BUN, Apache IV score, SOFA score, unittype. OR, odds ratio; CI, confidence interval; LRT, logarithm likelihood ratio test. * P<0.05 indicates that model II is significantly different from Model I. | | |

**Supplementary Table 4: Threshold effect analysis of the lactate and 28-day mortality**

| **Models** | **HR (95%CI)** | ***P*** value |
| --- | --- | --- |
| Model I | | |
| One line effect | 1.11(1.08, 1.15) | <0.0001 |
| Model II | | |
| Turning point (K) | 3.6 | |
| Lactate < K | 1.27 (1.13, 1.42) | <0.0001 |
| Lactate ≥ K | 1.09 (1.05, 1.13) | <0.0001 |
| P value for LRT test* |  | 0.021 |
| Data were presented as HR (95% CI) P value; Model I, linear analysis; Model II, non-linear analysis. Adjusted for age, gender, COPD, CHF, AMI, DM, heart rate, temperature, hemoglobin, potassium, RBC, sodium, WBC, bicarbonate, RDW, BUN, Apache IV score, SOFA score, unittype. HR, hazard ratio; CI, confidence interval; LRT, logarithm likelihood ratio test. * P<0.05 indicates that model II is significantly different from Model I. | | |

**Supplementary Figure 1**

Title: Associations between the lactate and 28-day mortality in elderly patients with sepsis.

**
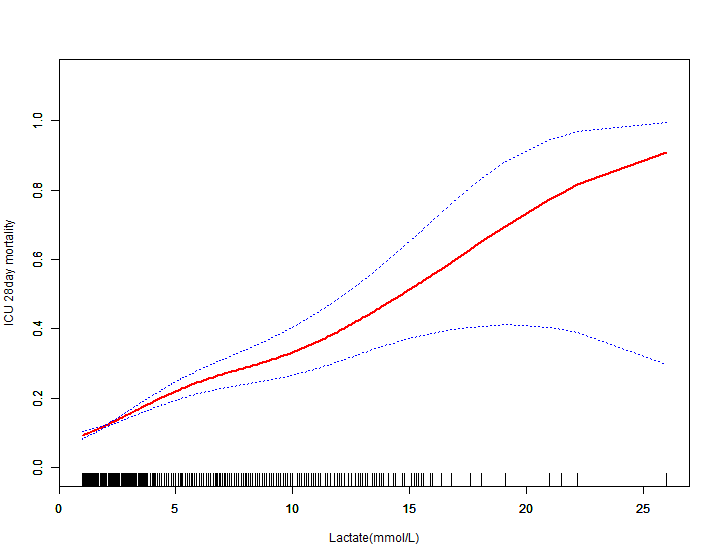
**

Note: A threshold, nonlinear association between the lactate and 28-day mortality was found in a generalized additive model (GAM) . Solid rad line represents the smooth curve fit between variables. Blue bands represent the 95% of confidence interval from the fit. Adjusted for age, gender, COPD, CHF, AMI, DM, heart rate, temperature, hemoglobin, potassium, RBC, sodium, WBC, bicarbonate, RDW, BUN, Apache IV score, SOFA score, unittype.

**Supplementary Figure 2**

Title: Associations between lactate and 28-day ICU mortality across different SOFA score categories.

**
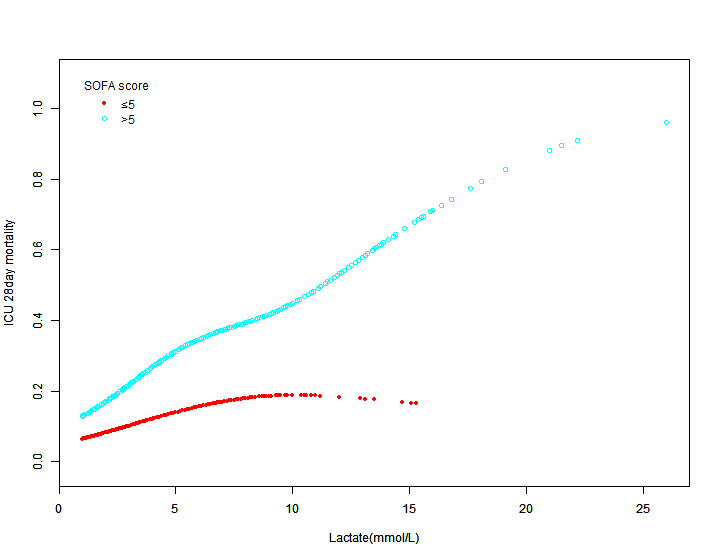
**

Note: The present study conducted a statistical analysis of the data with lactate levels as the exposure variable, ICU 28-day mortality as the outcome variable, and SOFA score as the stratification factor.

**Supplementary Figure3**

Title: Associations between the lactate and 28-day mortality in elderly patients with sepsis.

**
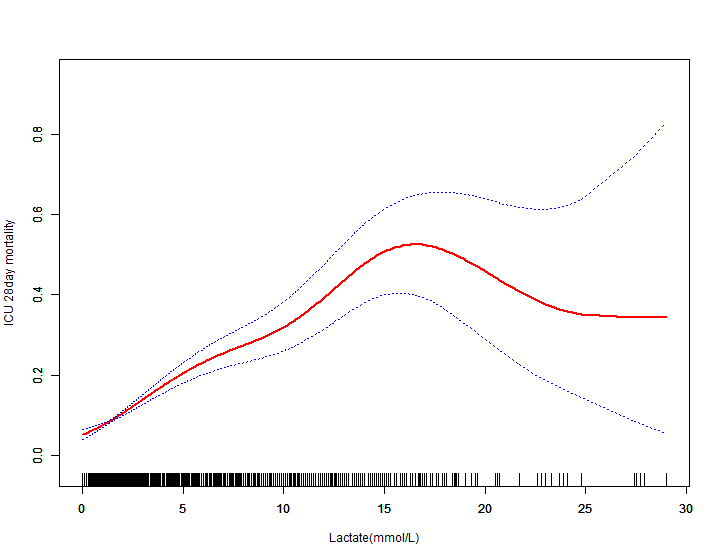
**

Note: A threshold, nonlinear association between the lactate and 28-day mortality was found in a generalized additive model (GAM) . Solid rad line represents the smooth curve fit between variables. Blue bands represent the 95% of confidence interval from the fit. Adjusted for age, gender, COPD, CHF, AMI, DM, heart rate, temperature, hemoglobin, potassium, RBC, sodium, WBC, bicarbonate, RDW, BUN, Apache IV score, SOFA score, unittype.

**Supplementary Figure 4**

Title: Associations between lactate and 28-day ICU mortality across different SOFA score categories.

**
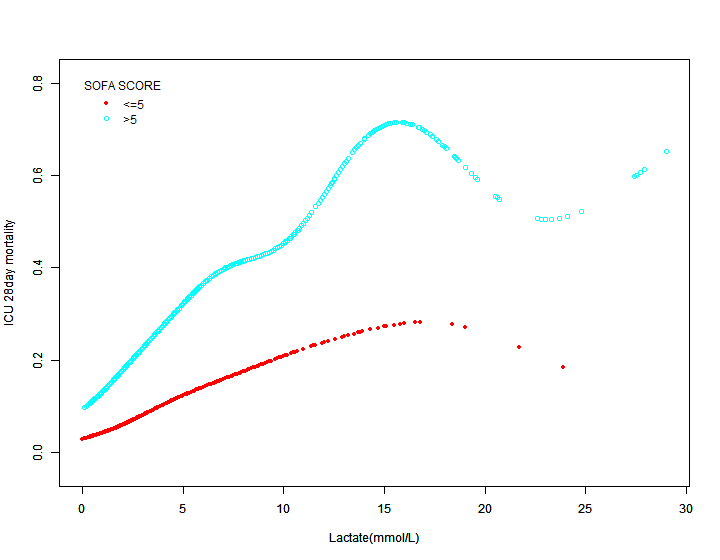
**

Note: The present study conducted a statistical analysis of the data with lactate levels as the exposure variable, ICU 28-day mortality as the outcome variable, and SOFA score as the stratification factor.

**Supplementary Figure5**

Title: Associations between the lactate and 28-day mortality in elderly patients with sepsis.

**
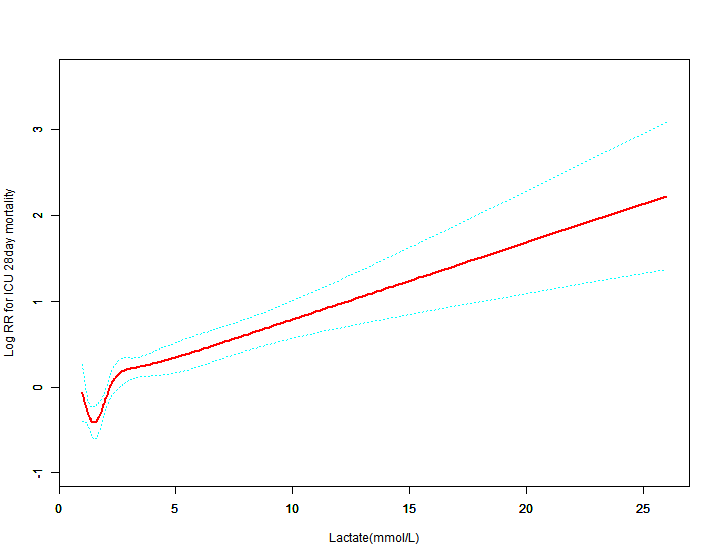
**

Note: A threshold, nonlinear association between the lactate and 28-day mortality was found in a Cox model with restricted cubic spline. Solid rad line represents the smooth curve fit between variables. Blue bands represent the 95% of confidence interval from the fit. Adjusted for age, gender, COPD, CHF, AMI, DM, heart rate, temperature, hemoglobin, potassium, RBC, sodium, WBC, bicarbonate, RDW, BUN, Apache IV score, SOFA score, unittype.

**Supplementary Figure6**

Title: Associations between lactate and 28-day ICU mortality across different SOFA score categories.

**
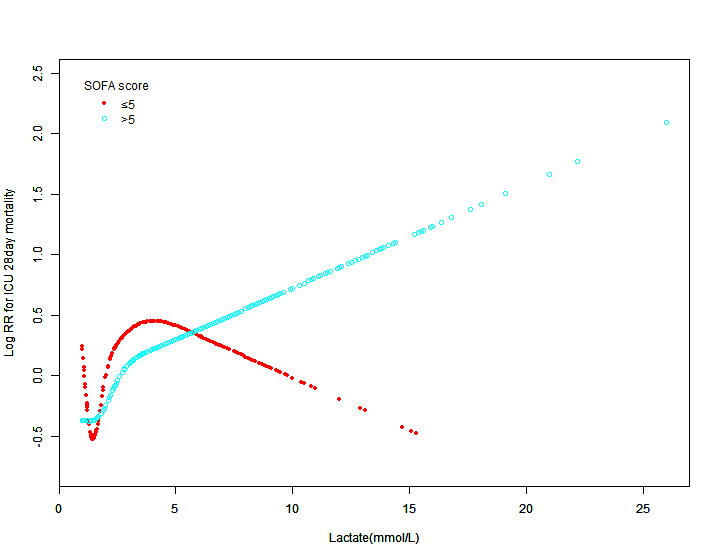
**

Note: The present study conducted a statistical analysis of the data with lactate levels as the exposure variable, ICU 28-day mortality as the outcome variable, and SOFA score as the stratification factor.
